# A comprehensive study of the academic benefits and practical recommendations to include resistance training programs in institutional education

**DOI:** 10.3389/fpsyg.2024.1387162

**Published:** 2024-05-16

**Authors:** Oscar Fraile-Martinez, Cielo García-Montero, Marcos Fraile-Martinez, Leonel Pekarek, Silvestra Barrena-Blázquez, Laura López-González, Miguel Ángel Álvarez-Mon, Tatiana Pekarek, Carlos Casanova, Melchor Álvarez-Mon, Raul Diaz, Miguel A. Saez, Miguel A. Ortega

**Affiliations:** ^1^Department of Medicine and Medical Specialties (CIBEREHD), Faculty of Medicine and Health Sciences, University of Alcalá, Alcala de Henares, Spain; ^2^Ramón y Cajal Institute of Sanitary Research (IRYCIS), Madrid, Spain; ^3^Department of Nursing and Physiotherapy, Faculty of Medicine and Health Sciences, University of Alcalá, Alcala de Henares, Spain; ^4^Surgery Service, University Hospital Principe de Asturias, Alcala de Henares, Spain; ^5^Department of Surgery, Medical and Social Sciences, Faculty of Medicine and Health Sciences, University of Alcalá, Alcala de Henares, Spain; ^6^Immune System Diseases-Rheumatology Service, University Hospital Principe de Asturias, Alcala de Henares, Spain; ^7^Pathological Anatomy Service, Central University Hospital of Defence-UAH, Madrid, Spain

**Keywords:** resistance training, academic performance, educational research, physical activity, university

## Abstract

The connection between physical activity and cognitive function has become a focus of attention in educational research in recent years. Regular exercise has been shown to have significant positive effects on physical health, but it also appears to have a significant impact on cognitive function and academic performance. Of all the exercise modalities, resistance training has drawn interest for its ability to improve cerebral abilities in addition to physical well-being. However, there is limited available knowledge exploring the relationship between resistance training regimens and academic performance. This narrative review aims to investigate the underlying mechanisms linking resistance training to academic performance. Firstly, we will examine the biological mechanisms and psychosocial links that potentially connect resistance training to academic performance to find and describe the different mechanisms by which resistance training improves academic performance. In the next part of the work, we delve into the existing observational and intervention studies that have explored the relationship between resistance training and academic performance. Lastly, we provide practical recommendations for including resistance training in institutional education settings, emphasizing the need of dispelling myths and addressing barriers to increase participation as well as the relevance of considering key training variables and adaptation of protocols to developmental stages, always guided by a properly trained professional. Overall, the available evidence supports that resistance training provides potential benefits to the academic performance of youth students with many biological and psychosocial factors that explain this relationship. However, most of the studies are observational, and broader interventional studies are needed to understand and maximize the benefits of this type of physical exercise.

## 1 Introduction

The concept of strength has different meanings depending on the context. If the idea is applied to sports education and the scope of the present study, it refers to the ability of a muscle to overcome external resistance. Strength is supported by several morphological and neural factors including muscle cross-sectional area and architecture, musculotendinous stiffness, motor unit recruitment, frequency encoding, motor unit synchronization, and neuromuscular inhibition (Suchomel et al., 2018). Resistance training consists of the introduction of exercises and movement patterns periodically that stimulate the muscle’s ability to exert force and combine stimulation of its hypertrophy (Phillips and Winett, 2010). Although resistance training has traditionally been related to the maximum amount of weight with which an individual can perform one repetition (1RM), other authors defend the need to include not only this element but also other variables such as the number of repetitions, the number of series, rest intervals between series, time under tension, order of exercises and critically, the effort perceived by each subject (Phillips and Winett, 2010). There are multiple practices that challenge the muscles and that can be considered resistance training, from the most basic requirements (Hollingsworth et al., 2020) to preparations that push the human body to the limit (Winwood et al., 2015). Within the various practices, bilateral training, accentuated eccentric training and loading, as well as variable resistance training, can produce the greatest comprehensive strength adaptations. However, bodyweight, isolation, plyometric, unilateral or kettlebell exercises, despite having less potential to improve maximal strength, are still relevant and truly useful for strength development, challenging the expression of strength in a limited time and differentially challenging motor demands (Suchomel et al., 2018).

Resistance training has multiple benefits for those individuals who practice it regularly. The relevance of including resistance training programs has been demonstrated in a wide variety of fields, whether for esthetic purposes (Rosenthal et al., 2021), to achieve specific physical performance (Ferland and Comtois, 2019) or even as a therapeutic approach (Montoro et al., 2015; Avilés-Martínez et al., 2022). Strength has been established as a direct indicator of health, contributing to an improvement in the quality of life and basic physical abilities (Celis-Morales et al., 2018; Alizadeh et al., 2023). The regular inclusion of resistance training programs improves lean body mass and exerts direct benefits on the musculoskeletal system, which is one of the pillars of maintaining acceptable standards of living in the individual (Bellido and Bellido, 2016; Nava-Bringas et al., 2018; Ortega et al., 2021). In fact, the musculoskeletal system is especially vulnerable to numerous pathologies and conditions, and may be a premature indicator to detect dysfunction in the body (Bhimani et al., 2021; Damluji et al., 2023). On the other hand, resistance training also exerts notable benefits on the different organs and systems of the body, as well as on mental, emotional and social well-being (Maestroni et al., 2020). Brain and muscle are connected through different mechanisms by the denominated muscle-brain axis (Burtscher et al., 2021; Rai and Demontis, 2022). A growing body of evidence supports that the mind-muscle connection during exercise is essential not only for progressing in the training programs (Calatayud et al., 2016), but also for maximizing the cognitive benefits of physical activity in the brain (Blomstrand et al., 2023). Therefore, scientific evidence conclude that resistance training is essential not only for ensuring a global health of the subject, but also for maximizing mind and brain functioning.

In the pursuit of academic excellence, both students and educators continually seek innovative strategies to improve cognitive skills and optimize learning outcomes. The predominant perception of physical exercise as a means to improve cardiovascular health and muscular strength has undergone a paradigm shift in recent years. While traditional approaches have focused primarily on intellectual exercises such as studying, reading, and problem-solving (Azer et al., 2013), emerging research has highlighted the potential impact of physical activities, such as endurance, flexibility, or resistance training, on cognitive function and academic performance (Trudeau and Shephard, 2009). Whereas various observational and interventional studies have provided initial evidence of an association between resistance training and academic performance, there is a lack of comprehensive studies analyzing the different interconnections, the relevance and perspectives from including regular resistance training to enhance academic performance in institutional education. This narrative review delves into the promising relationship between resistance training and academic performance, intending to uncover the underlying mechanisms that connect both factors while analyzing the available literature and offering practical recommendations to include resistance training programs in institutional settings.

The primary research question guiding the investigation into the relationship between resistance training and academic performance is:

- What are the underlying links between resistance training and academic performance?

Subsequently, the inquiry will be divided into three main points exploring different aspects of these links.

Firstly, to describe the link between resistance training and academic performance we aimed to respond the following question:

- What are the specific biological and psychosocial mechanisms underlying the relationship between resistance training and academic performance?

Secondly, to analyze the available studies connecting resistance training and academic performance we focus on the following questions:

- What evidence from observational studies supports the relationship between resistance training and academic performance and what findings have intervention studies revealed about the impact of resistance training on academic performance?

Lastly, to offer practical recommendations for including resistance training programs in institutional settings we propose the following questions:

- What key training variables should be considered when implementing resistance training programs by proper professional trainers in educational settings?
- How can resistance training protocols be adapted to different developmental stages and individual factors like sex to optimize academic performance?

## 2 Resistance training and academic performance—what are the links?

Physical education as a teaching and training instrument has its origins in Greek *paideia*, an educational ideal in which both cultural and physical instruction as a whole was valued. In fact, during the Hellenistic era, the concept of “citizen” was not conceived without athletic preparation carried out in the gym during his adolescence (Alonso-Troncoso, 2009). Within the educational landscape, the importance of promoting not only cognitive skills but also general well-being is increasingly recognized (Haapala, 2022). Thus, integrating physical activity, specifically resistance training, into academic settings is a promising strategy not only to improve physical health but also to potentially elevate cognitive abilities essential for academic performance. As it will be herein explored, resistance training promotes a broad range of biological and psychosocial effects potentially related to academic performance. In this section the main mechanisms that explain the association between resistance training and academic performance will be described.

### 2.1 Biological mechanisms

To understand the biological effects of resistance training, it is essential to highlight that physical exercise in general operates as a hormetic agent. In other words, it acts as a stressor in the body that follows a biphasic dose–response curve inducing a series of adaptations. Progressive and beneficial in the body as long as a minimum threshold is reached, and a maximum threshold is not exceeded (Mattson, 2008). Thus, the maximum benefits of physical activity in general and resistance training are observed at a certain dose, with both its deficiency and excess being associated with negative results (Peake et al., 2015). However, the ideal resistance training dose where the maximum benefits will be reported for each person will vary depending on their characteristics and a series of factors that will not be covered below, entering into important variables such as personalization and adaptation of the training to each person. In this section we will consider that the individual receives an optimal dose of exercise without considering these details through which the described mechanisms exert a favorable effect, and its impact, from a general perspective, at the level of academic performance.

In this context, it is known that there are multiple ways in which the two factors can be interconnected. It is important to understand that this type of physical activity exerts direct biological effects on the brain, regulating the production of certain neurotransmitters and neuromodulators, as well as indirectly through its actions in the different tissues of the body (Maestroni et al., 2020). For example, the modulatory effect of resistance training has been described on a large number of neuromodulators related to academic performance such as lactate, brain-derived neurotrophic factor (BDNF), insulin-like growth factor 1 (IGF-1), vascular endothelial growth factor (VEGF), acetylcholine, dopamine, norepinephrine and serotonin (Basso and Suzuki, 2017). The regulation of these neurochemical agents is translated and accompanied by structural and functional changes in different areas of the brain (neuroplasticity) (Silverman and Deuster, 2014), in the renewal and formation of new neurons (neurogenesis) (Azevedo et al., 2023), in increasing cerebral blood flow (Cheng et al., 2022) leading to positive improvements in mood, behavior and cognitive functioning (Basso and Suzuki, 2017). According to previous works, these brain changes related to physical activity among other factors has shown a positive correlation with academic performance (Alghadir et al., 2020), thus demonstrating the potential benefits from resistance training in this field.

On the other hand, resistance training regulates the activity of different neuroendocrine axes such as the hypothalamus-pituitary–adrenal, hypothalamus-pituitary-somatotropic and hypothalamus-pituitary-gonadal (Cano Sokoloff et al., 2016; Anderson et al., 2019), whose relevance in academic performance has been demonstrated in previous studies (Martin and Steinbeck, 2017; Alghadir et al., 2020; Hinds and Sanchez, 2022). On the other hand, we must also highlight the important benefits of resistance training on a systemic level. In more detail, resistance training directly modulates the production of so-called organokines, endocrine products produced and released by the muscle (myokines), adipose tissue (adipokines), the liver (hepatokines) or the intestine that orchestrate and regulate several metabolic and immunoinflammatory processes (Lőrincz et al., 2023). In more detail, resistance training seems to improve insulin sensitivity and has positive effects on weight control and different variables, especially in children and adolescents with overweight or obesity (Kazeminasab et al., 2023). The association between obesity and academic performance is a topic of great relevance, due to an uninterrupted increase in the prevalence of pediatric obesity during recent decades (Jebeile et al., 2022). In fact, a negative association between obesity and academic results has been established in 32 countries studied, after controlling for confounding factors. In more detail, it is known that children with a healthy weight are 13% more likely to perform well in school than children with obesity (Devaux and Vuik, 2019), although the multifactorial dimension of this entity resulting from the interconnection of biological, social, cultural, psychological, educational and individual factors must be understood and addressed as a whole. Regarding the immunological effects of resistance training, available literature has shown that acute and chronic resistance training is able to induce several changes in the immune system, being the latter the most favorable to improve immunity and reduce systemic inflammation (Fortunato et al., 2018; Salimans et al., 2022). Likewise, it is worth highlighting the favorable effect of resistance training on the diversity and functionality of the gut microbiota both in healthy subjects and in individuals with different clinical pathologies (Boytar et al., 2023). In this sense, previous works have shown the direct association between metabolic health, and a proper balance of the gut microbiota and the immune system in the academic performance of young people (Esteban-Cornejo et al., 2016; Holmes et al., 2018; Lapidot et al., 2023), thus demonstrating that physical exercise in general and resistance training can critically benefit individuals trained from these mechanisms in the educational system.

The benefits of resistance training on bone health are also well known to everyone, having a direct and positive impact not only on older people or people at risk of bone pathology but also on children and adolescents. Unfortunately, it has been reported that a large number of children and adolescents (particularly adolescent girls) do not meet the recommended 60 min of physical activity per day and, especially, with adequate bone loading stimulation to optimize bone accumulation and health (Faigenbaum et al., 2009). Although to our knowledge a direct relationship between bone health and academic performance has not been demonstrated, the functionality of this organ is essential for the health of elderly individuals and young subjects, which eventually influences academic performance (Kohl and Cook, 2013). Likewise, resistance training has very positive effects on the cardiovascular system, which is directly associated with better academic results according to different studies, systematic reviews and meta-analyses (Sardinha et al., 2016; Álvarez-Bueno et al., 2020; Ishihara et al., 2021). Finally, habitual physical activity and resistance training are commonly related to other lifestyle variables like proper nutrition and sufficient sleep, whereas sedentarism, an unbalanced diet and inadequate resting leads to different physical and mental concerns (Fraile-Martinez et al., 2022). By controlling these variables and others like (ab)use of screens or the consumption of toxic substances, resistance training can have an enormous and synergistic impact with these factors on the academic performance of young people (Sánchez-Hernando et al., 2021), demonstrating the necessity to encourage and integrate resistance training into a healthy lifestyle to maximize its benefits.

**Figure 1:**
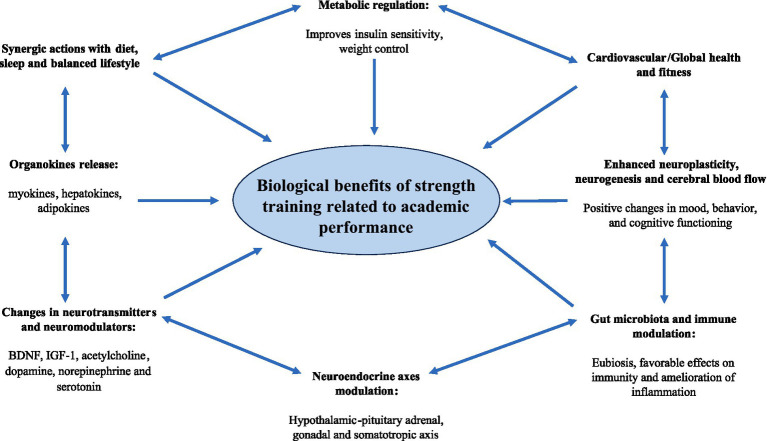
Biological effects of resistance training related to academic performance.

The different biological effects of resistance training potentially involved in academic performance are detailed in Figure 1.

### 2.2 Psychosocial links

Resistance training is also closely linked to a series of psychological and social effects that may be involved in improving students’ academic performance. One of the most notable effects of resistance training is its ability to favorably influence the individual’s emotional management and mental health. Among other effects, habitual resistance training has been associated with both anxiolytic and antidepressant effects (Strickland and Smith, 2014; Gordon et al., 2018), and as mentioned previously, there is a clear relationship between resistance training and better management and reduction of stress perceived by students who practice it regularly (Becker et al., 2021). The benefits of resistance training on mental health are due to its biological effects described above and the improvements in different psychosocial domains that will be addressed in this section. Current scientific literature supports the clear and worrying inverse association between growing mental health problems and academic performance in children, adolescents and young people, mainly due to alterations in different cognitive domains or absenteeism that the affected subjects present (Agnafors et al., 2021; Jeffries and Salzer, 2022). In this sense, resistance training represents a key element that should be considered for the best academic performance of students, especially in those groups more vulnerable to stress or with signs of mental health problems.

A fundamental psychological consequence reported from resistance training has to do with the improvement of self-confidence and self-esteem of the subject who practices it, not only on a physical level but also extrapolated to other areas of life (Collins et al., 2019). Past studies (Bartholomew et al., 2011) have shown how 12 weeks of scheduled resistance training is associated with improvements in different domains of strength, which promotes an improvement in the subject’s physical self-perception and general self-esteem. Likewise, resistance training, separately or in combination with aerobic training, also significantly improves self-esteem and self-perception in overweight or obese young people, these benefits being superior to aerobic training alone (Goldfield et al., 2015). The importance of self-confidence and self-esteem in academic performance is supported by the available scientific literature (Mathew, 2017). Therefore, the benefits of exercise in this psychological domain is another critical point to explain the observable relationship between resistance training and academic performance.

The direct benefits of resistance training in enhancing social skills are also a key factor considered in the scientific literature. In more detail, carrying out collective activities in different areas (including physical or resistance training) has a favorable translation toward improvements in team work and performance (Salas et al., 2008; Mcewan et al., 2017). Likewise, other studies have found that subjects who train strength and perform regular physical activity tend to have more prosocial behaviors and exhibit more confidence than those who do not train (Di Bartolomeo and Papa, 2019). The enhancement of social skills and teamwork has many benefits in young people, having shown how interventions that improve these domains have a very positive impact on academic performance, as supported by recent scientific evidence (De Prada et al., 2022).

On the other hand, studies have also observed a relationship between resistance training has been linked to improved attention, concentration and working memory, together with enhanced language and arithmetic skills (Haapala, 2022), thus affecting academic performance. For example, the inclusion of strength and speed programs in children showed a considerable improvement in the Numbers Test when compared to those who did not perform such a training program (Polevoy, 2022). These benefits of resistance training in attention may be especially important in vulnerable children and adolescents who have conditions such as attention deficit hyperactivity disorder (ADHD), also improving their sociability, motor skills and neuropsychological parameters (Kamp et al., 2014). According to a systematic review and meta-analysis (Robinson et al., 2023), the inclusion of strength programs has shown a favorable association between the improvement of attention and of other cognitive domains (cognitive flexibility, inhibition, working memory, planning and fluid intelligence) with academic performance in children and adolescents, although more studies are still required to establish more conclusive results.

Discipline, perseverance, and the generation of study and healthy living habits are essential to maximize students’ academic performance (Simba et al., 2016; Innocent and Opiyo Andala, 2021). Previous work has shown how resistance training, total physical activity, a low body mass index (BMI) and the intake of healthy foods are positively associated with academic performance, while few hours of sleep, consumption of ultra-processed foods, physical inactivity, high BMI, and substance abuse have a negative relationship with it (Kristjánsson et al., 2010; Reuter and Forster, 2021). Although these associations are clearer in children and adolescents, the impact that these habits or BMI itself may have on the academic performance of university students seems to be less evident, making it necessary to conduct more studies that evaluate the impact of regular resistance training on this group (Calestine et al., 2017). Similarly, resistance training also helps with planning, problem-solving, and executive function, with these facts having a favorable impact on school performance (Robinson et al., 2023). Finally, the inclusion of physical training programs in children and adolescents promotes important improvements in enjoyment, perceived autonomy, intrinsic motivation, self-determination, and task completion by students, thus having a direct effect on academic performance (Kelso et al., 2020).

**Figure 2:**
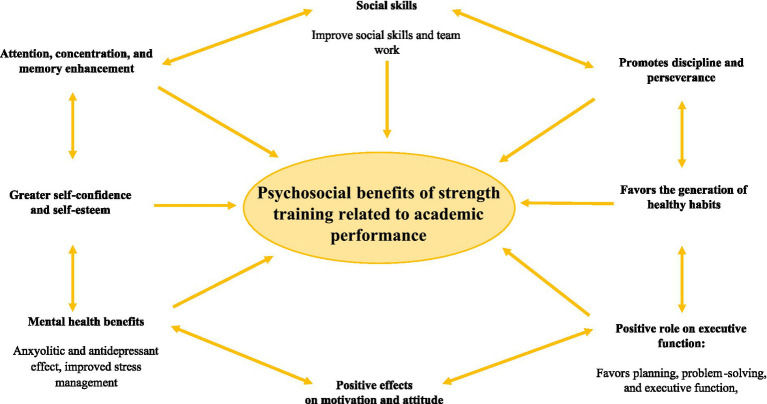
Psychosocial effects of resistance training related to academic performance.

Figure 2 details the relationship of resistance training with the psychosocial benefits associated with the academic performance of students.

## 3 Resistance training regimes for improving academic performance

In this section, the current literature that exists around the implementation and relation between resistance training with academic performance will be assessed. First, the main studies that detail the effects of resistance training on academic improvement will be collected, although the vast majority of them are observational rather than interventional. Then, important specific considerations and recommendations will be made in order to include resistance training regimes considering multiple training variables according to available literature.

### 3.1 Observational and intervention studies connecting resistance training with academic performance

Various interventional studies have found a potential causal role between promoting exercise and resistance training with academic performance, whereas the vast majority of the studies are only observational. However, and as, it will be later remarked, an adequate programming and supervision of resistance training is essential for maximizing their results and ensuring the safety in young subjects. Although everyone can benefit from resistance training, there are important variables that can determine the extent to which its implementation can be effective, such as gender, age, or the existence of different underlying conditions or circumstances. For instance, Cappelen et al. (2017) reported how encouraging and promoting gym attendance leaded to a significant increase of total grade points of 0.15 standard deviations of 400 college students when compared to a control group of 382 students. Intriguingly, these effects were 2-fold greater if the student had struggled with lifestyle before the intervention. Similarly, studies have observed that male students with greater strength and cardiopulmonary capacity were those who obtained better academic performance, as well as female students who had greater flexibility, strength, endurance and cardiopulmonary capacity (Kuo-Ming et al., 2012). Thus, the authors observed how girls can benefit from more types of training, and not only that, but they also reported how physical skills boosted academic performance twice as much in their case as in boys. Other studies have also found similar differences in the association between physical fitness and academic performance according to gender. Van Dusen et al. (2011) observed that out of 254,743 school student records, the associations between cardiovascular fitness and academic performance were stronger in girls than in boys, particularly in reading and math outcomes. Thus, it could be concluded that in general females benefit more clearly from their physical fitness in academic performance as well as from different types of training, while in men, although they also benefit, they do so in a less strong manner and more focus on strength. However, regarding the implementation of RT programs, the literature supports that females can also benefit from this type of interventions through improving their self-acceptance, personal growth, flow state mind, social affiliation and autonomy (Hall and Noonan, 2023), which eventually as previously stated improve academic performance. Overall, scientific evidence seems to indicate that although there are sex-related differences across males and females, the inclusion of RT programs has a great impact for both groups, and even it could be greater for women, as they often are less engaged in strengthening-related activities.

On the other hand, it seems that age may play a certain role in the assimilation of resistance training and its relationship with academic performance; however, the available evidence in this regard is highly heterogeneous. On the one hand, there are studies that have shown a direct association between strength tests and academic performance in children between 9 and 12 years old, observing more significant effects in older boys and girls (Du Toit et al., 2011). On the other hand, other works (Gouveia et al., 2020) have shown how those students with better physical fitness (including functional strength, among other parameters) showed a greater improvement in academic performance at a 1-year follow-up, with this effect being magnified in younger students. In fact, they found that the interaction of physical fitness with age predicted 45.7% of the variance in the change in academic performance. In any case, the evidence shows a direct association between strength levels and academic performance even in university students. In this sense, Keating et al. (2013) observed in university students a direct relationship between the number of weekly strength sessions they performed with the average grades they obtained. Similarly, Peña et al. (2019) also reported a direct association between maximum strength and academic performance in university students. Thus, it can be concluded that although age may be an important factor to understand how much resistance training affects academic performance, its benefits are observed both at school and at college and university, therefore, the support of these institutions in the regular performance of resistance training for their students.

The implementation of resistance training can even magnify its effects in more vulnerable subjects or with different conditions. For example, and as mentioned previously, overweight and obese students can especially benefit from the introduction of these programs, as demonstrated by Cadenas-Sanchez et al. (2020). After evaluating a total of 106 overweight/obese children, they observed how field-based cardiorespiratory fitness was associated with linguistic skills, field-based muscular strength with grade point average, natural and social sciences, and foreign language; Speed-agility was correlated to some language-related skills and the laboratory-based muscular strength presented an association with mathematical skills. However, they found that the effects of muscular strength and speed/agility were attenuated and disappeared in many cases after additional adjustments for body mass index and cardiorespiratory fitness, concluding that these variables were interdependent. Resistance training (especially conventional versus other types of interventions) has also demonstrated notable benefits in improving academic performance in children and adolescents with psychosocial disorders (Barahona-Fuentes et al., 2021). There are also studies that have shown how levels of physical fitness and strength serve to discriminate between students with low and high academic performance (Du Toit et al., 2011; Keating et al., 2013). thus suggesting how these students could benefit even more from these interventions. Similarly, Smail and Horvat (2006) have also observed how community- and school-based strength programs improve physical functioning and work-related skills. in subjects with intellectual disability, being further proof that the most vulnerable individuals can show great benefits by improving their strength levels.

Collectively, while there is evidence to suggest a positive association between physical activity and strength with academic performance, more intervention studies specifically targeting resistance training and academic performance are needed. In Table 1, main results collected in our study are summarized.

**Table 1:** Summary of the main intervention and observational studies directly relating resistance training with academic performance.

Reference	Observational/Interventional	Population group	Association observed between resistance training and academic performance
Cappelen et al. (2017)	Interventional	400 college students vs. 382 control group from the University of Bergen and the Bergen City College. The average age was of 22 years old with approximately 50% of female and 50% of male. Lifestyle index, study hours and happiness was not different at baseline between groups.	Significant increase in total grade points (0.15 standard deviations) in students encouraged for gym attendance. Effects 2-fold greater for those struggling with lifestyle before the intervention.
Kuo-Ming et al. (2012)	Observational	1,065 children from fourth to sixth grade from Taiwan (Male 49.58%; female 50.42%) Fourth grade: Girls: 10.05 ± 0.28 years, Boys: 10.04 ± 0.30 years Fifth grade: Girls: 11.08 ± 0.30 years, Boys: 11.14 ± 0.35 years Sixth grade Girls: 12.09 ± 0.30 years, Boys: 12.10 ± 0.30 years	Male students with greater strength and cardiopulmonary capacity had better academic performance. Female students with greater flexibility, strength, endurance, and cardiopulmonary capacity showed better academic performance. Physical skills boosted academic performance twice as much in girls compared to boys.
Van Dusen et al. (2011)	Observational	254,743 Texas public school students classified from 3 to 11 grade Level (48.7% male /51.3% female)	Associations between cardiovascular fitness and academic performance were stronger in girls, particularly in reading and math outcomes. Females generally benefit more clearly from physical fitness in academic performance.
Hall and Noonan (2023)	Observational	10 women from 18 to 27 years old undergoing RT programs	Females can benefit from RT interventions through improvements in self-acceptance, personal growth, flow state mind, social affiliation, and autonomy, positively impacting academic performance.
Du Toit et al. (2011)	Observational	212 children from South Africa (94 boys, 118 girls) with ages comprised between 9–12 years old	Direct association between strength tests and academic performance, more significant effects according to age in boys and girls.
Gouveia et al. (2020)	Observational	142 pupils from the 5th to the 12th year from Madeira (Portugal) (Median age = 14.59 years, 50% boys, 50% girls)	Students with better physical fitness, including functional strength, showed a greater improvement in academic performance at a 1-year follow-up, with effects magnified in younger students. Age interacts with physical fitness, predicting variance in academic performance.
Keating et al. (2013)	Observational	Health behavior data (N = 1,125) collected by the American College Health Association at the university in 2008 (61.5% of them were female). The average age was of 22.21, including whites (57.0%), Asians (20.5%), and Latinos (17.2%) students. 95.1% of participants were full-time students	Direct relationship between the number of weekly strength sessions and average grades obtained in university students.
Peña et al. (2019)	Observational	135 students of the fifth semester of the Areandina University in Bogota, Colombia (average age 21.8 ± 4.5 yr.; 70.3% male versus 29.7% female)	A positive correlation was established between maximum strength and academic performance.
Cadenas-Sanchez et al. (2020)	Observational	106 overweight/obese children (57.5% boys, 43.5% girls).	Cardiorespiratory fitness, muscular strength and speed-agility are positively associated with academic performance. Although these associations appear to be interdependent on body mass index and cardiorespiratory fitness.

### 3.2 Practical recommendations for including resistance training in institutional education

Although many studies focus on the effects of resistance training in elderly subjects and adults with or without pathologies, the introduction of resistance training programs in children and adolescent populations has experienced exponential growth in recent years (Pochetti et al., 2018). It is important to highlight that there are still certain myths and misunderstandings regarding resistance training in children and adolescents, particularly regarding its safety, negative effects on development and other points that still represent an important barrier to break down (Brooks et al., 2022). Indeed, it is possible that this could be one reason of the low proportion of intervention studies relating resistance training with academic performance. Likewise, family situation or purchasing power can considerably affect the initiation of a child or adolescent into strength programs (Paredes et al., 2021). The teaching and introduction of basic principles of physical conditioning carried out at an early age, regardless of the context, can entail a series of advantages on a physical and psychological level, which can improve the student’s academic performance as previously mentioned. However, the short duration imposed on it is not commensurate with the multiple benefits it is capable of providing to the physical and mental health of the child or adolescent. Thus, in many cases, the physical education taught in educational centers is the only direct contact the child/adolescent has with sport and unfortunately the number of hours taught in this subject is usually 1.5 to 3 h per week (García-Baños et al., 2020), figures much lower than the recommendations of moderate-vigorous physical activity at least 1 h a day and musculoskeletal strengthening 3 or more times a week (Landry and Driscoll, 2012).

The protocol to follow and the control of the different training variables (including the exercises performed, the order, the series, the repetitions, the load, the contractions, the intensity of the effort and the volume of strength) represent key pillars of the research in this field (Fisher, 2013). According to a systematic review, study duration and training volume are variables directly correlated with favorable results in resistance training studies in children and adolescents (Behringer et al., 2010). On the other hand, the intensity and recovery times between exercises are also important resistance training variables in this group. According to the literature (Lloyd et al., 2014), training intensity and volume are two inverse and complementary variables that must be balanced to minimize the risk of injury, due to poor technique. and execution of the exercise (excess intensity) or over training (excess volume). In fact, injuries that have been reported in resistance training studies in this population have been attributed to misuse of equipment, excessive weight, inefficient technique, and lack of supervision by qualified adults (Dahab and McCambridge, 2009). Thus, it is essential that resistance training is carried out under adequate supervision and programming to provide maximum benefits to young people.

According to studies and competent authorities, there is no recommended minimum age to start RT programs in young people (Behm et al., 2008). However, training and instruction should be appropriate for this group of individuals, and involve a warm-up, cool-down, and appropriate exercise choice. With respect to training in younger children, the main objective proposed is to create a series of customs and practices in the childhood, without going into so much detail in physical conditioning. In accordance with the bibliography could it would be interesting to introduce any type of physical activity that allows increasing the activity factor, thus avoiding harmful habits such as a sedentary lifestyle, which can be one of the main factors of obesity during adolescence and youth (Fisberg et al., 2016). On the other hand, it is known that children’s ability to gain muscle mass increases proportionally with age and maturity, and although it does not seem to have a clear association with the onset of puberty (Behringer et al., 2010). Thus, the research carried out in this field reminds that training must be designed appropriately for the emotional and developmental stage of the participant for their safety, and not only their chronological age (Myers et al., 2017).

Regarding resistance training in children and adolescents, it would be interesting to focus first on movements and practices inherited from calisthenics and gymnastics. These linked disciplines have numerous advantages. Apart from the proper effects on strength, it promotes other physical benefits such as flexibility, body control and agility (Desai et al., 2019) as well as muscular endurance (Mear et al., 2022), while presenting a low injury rate (Hart et al., 2018). Regarding the facilities necessary for its implementation, it is worth highlighting the few requirements for both infrastructure and material to carry them out. To give some examples, exercises such as abdominal crunch, different variations of jumping, push-ups, handstands or squats could be implemented. As the academic year progresses, the introduction of more complex exercises such as pull-ups, Nordic curls and parallel dips could begin to be considered, as well as introducing elements such as elastic bands, rings or suspension straps (TRX). The estimated training volume according to scientific evidence (Behm et al., 2008), could be around 2–3 times/week on non-consecutive days, with 1–2 sets Initially, progressing to 4 sets of 8–15 repetitions for 8–12 exercises.

As the child or adolescent progresses and master the different movement patterns based on the management of their own body weight, the strength program should adapt and evolve. The focus could be directed at a global activation of the muscles, with all the benefits that this entails and that was previously mentioned, including avoiding poor posture and achieving optimal muscle balance (Villarrasa-Sapiña et al., 2018). The training methodology should opt for the introduction of basic movement patterns. Among them, they would mainly highlight hip dominants, knee dominants, as well as both vertical and horizontal pushes and pulls. For example, exercise like shoulder press, chest press, rows, pulldowns, squats or deadlifts. Then, the introduction of new elements such as dumbbells or bars could be considered, but maintaining the basis of the training in your own body weight, elastic bands and suspension straps (Myers et al., 2017). However, it is essential to start the young person with exercises that involve all the main muscle groups with a relatively light weight, one to three sets of 6 to 15 repetitions, maintaining the 2 to 3 non-consecutive days per week. As more experience is gained, it is recommended to gradually increase the loads and add multi-joint exercises (Miller et al., 2010). Advanced movements such as Olympic-style lifting, plyometrics and balance training are also recommended, including different approaches such as simple power or strength exercises as well as strength-endurance and strength-power exercises (Behm et al., 2008; García-Baños et al., 2020). Children show a capacity to recover from rapid fatigue, with 1 min of rest between sets being sufficient for most of them and up to 2–3 min when the training intensity is increased (Lloyd et al., 2014). To adapt the intensity of the training to the programming, the estimation of measurements such as 1RM can be especially useful if you have the necessary materials for it; Otherwise the measurement of grip strength, vertical jump or long jump have been shown to have a direct relationship with 1RM in children and adolescents (Lloyd et al., 2014). Each session of Exercise should be adequately supervised for safety and to provide feedback on technique and form, regardless of the adolescent’s resistance training experience.

On the other hand, it would also be interesting to consider gender when it comes to personalized physical training programming, due to the differences discussed above. This circumstance becomes especially relevant during puberty, where sexual dimorphism is accentuated. During late adolescence and youth there is a tendency to individualize the study and consider the sexes as important training variables (Ramírez-Vélez et al., 2019). Regarding resistance training, women have demonstrated a superior capacity for muscle and hormonal recovery between sessions (Judge and Burke, 2010), being able to tolerate a greater number of effective repetitions and weekly volume (Hunter, 2014, 2016). In contrast, men generally have greater ease with high loads and greater absolute strength and power relative to differences in body mass, lean body mass, and muscle thickness between men and women (Bartolomei et al., 2021). Likewise, although musculoskeletal growth and development show very similar trends between genders, male and female strength and (neuromuscular) coordination patterns diverge significantly during and after puberty, with boys being the ones who naturally show an increase in power, strength, and body coordination with chronological age while untrained girls, on average, show little improvement in these domains during puberty (Faigenbaum and Myer, 2010). Also previous works have found that in general, more men than women met recommendations for muscle-strengthening activities in different countries (Nuzzo, 2020), and despite the participation rate was relatively low for both sexes, they evidenced the gender-related disparities for RT among men and women. As the literature support (Hurley et al., 2018), this gap is an important barrier to overthrow, especially if we consider that the benefits and effects of the same RT programs in men and women are quite similar and even women appear to receive more benefits and greater gains in relative strength, particularly in the upper body of untrained subjects (Roberts et al., 2020). Therefore, considering gender differences seems to be important for youth training, also considering other relevant factors such as physiognomy, socioeconomic context or adherence must also fit with individualized programming.

Finally, despite not specifically focused on academic performance, past works have remarked the association between resistance training and improvements in cognitive domains, which may indirectly impact on academic performance. For instance, the inclusion of a 10 weeks programs based on calisthenics and gymnastic exercises on children between 4 to 6 years old leaded to significant improvements in attentional networks in this group (Wick et al., 2021). Likewise, a systematic review collecting 36 randomized clinical trials (4,577 students) observed that acute exercises significantly improved working memory, inhibitory control, and cognitive flexibility in children and adolescents (Liu et al., 2020). High versus low/moderate intensity physical exercises might have differential biological and cognitive effects especially considering time course; although both types of trainings lead to improvements in cognitive functions (Brush et al., 2016; Hötting et al., 2016). The inclusion of physical training in real-world settings also seems to have significant benefits on cognitive improvement in children and adolescents (Shi et al., 2022). Globally, there is still a lack of evidence to make specific recommendations or aid in the selection of exercises that could bring the greatest benefits for cognitive improvement and other variables impacting academic performance in children and youths. Generating adherence to resistance training and an adequate programming personalized to age and individual factors might probably be the most important points to consider in these populations.

## 4 Conclusion

Throughout this work, we have tried to collect the available scientific evidence that connects the inclusion of resistance training programs with the academic performance of young students. The present narrative review aims to provide a comprehensive investigation integrating insights from various disciplines such as exercise science, psychology, and education. By exploring biological mechanisms, psychosocial factors, observational and intervention studies, our main objective is to provide and defend a holistic understanding of the relationship between resistance training and academic performance. Moreover, our study also intends to be practical, presenting actionable recommendations for implementing resistance training programs in educational settings. Overall, resistance training can influence in a very positive way through different mechanisms the academic performance of children, adolescents and university students, although there are certain groups that can benefit to a greater degree from its implementation, such as untrained subjects (especially females) or people with psychosocial disorders, overweight or obesity. The implementation of these programs seems to be useful in students of different ages, and the integration of multiple variables in future studies is also necessary to establish a clear causality between strength and academic performance.

Likewise, there are multiple questions still to be answered, mainly around which protocols can bring the maximum benefits to students or in what context it should be carried out, considering its inclusion with other types of training such as aerobic and flexibility training, whose benefits in the academic performance have also been demonstrated.

## Author contributions

OF-M: Conceptualization, Data curation, Formal analysis, Funding acquisition, Investigation, Methodology, Project administration, Resources, Software, Supervision, Validation, Visualization, Writing – original draft, Writing – review & editing. CG-M: Conceptualization, Data curation, Formal analysis, Funding acquisition, Investigation, Methodology, Project administration, Resources, Software, Supervision, Validation, Visualization, Writing – original draft, Writing – review & editing. MF-M: Conceptualization, Data curation, Formal analysis, Funding acquisition, Investigation, Methodology, Project administration, Resources, Software, Supervision, Validation, Visualization, Writing – original draft, Writing – review & editing. LP: Writing – original draft, Writing – review & editing. SB-B: Conceptualization, Investigation, Methodology, Writing – original draft, Writing – review & editing. LL-G: Writing – original draft, Writing – review & editing. MiÁ-M: Writing – original draft, Writing – review & editing. TP: Writing – original draft, Writing – review & editing. CC: Writing – original draft, Writing – review & editing. MeÁ-M: Writing – original draft, Writing – review & editing. RD: Writing – original draft, Writing – review & editing. MS: Writing – original draft, Writing – review & editing. MO: Conceptualization, Data curation, Formal analysis, Funding acquisition, Investigation, Methodology, Project administration, Resources, Software, Supervision, Validation, Visualization, Writing – original draft, Writing – review & editing.

## References

[ref1] Agnafors S. Barmark M. Sydsjö G. (2021). Mental health and academic performance: a study on selection and causation effects from childhood to early adulthood. Soc. Psychiatry Psychiatr. Epidemiol. 56, 857–866. doi: 10.1007/S00127-020-01934-5, PMID: 32813024 PMC8068628

[ref2] Alghadir A. H. Gabr S. A. Iqbal Z. A. (2020). Effect of gender, physical activity and stress-related hormones on Adolescent’s academic achievements. Int. J. Environ. Res. Public Health 17, 1–14. doi: 10.3390/IJERPH17114143, PMID: 32531964 PMC7311984

[ref3] Alizadeh S. Daneshjoo A. Zahiri A. Anvar S. H. Goudini R. Hicks J. P.. (2023). Resistance training induces improvements in range of motion: a systematic review and Meta-analysis. Sports Med. 53, 707–722. doi: 10.1007/S40279-022-01804-X, PMID: 36622555 PMC9935664

[ref4] Alonso-Troncoso V. (2009). The Hellenistic gymnasium and the pleasures of paideia. Symb. Philol. XIX, 71–84.

[ref5] Álvarez-Bueno C. Hillman C. H. Cavero-Redondo I. Sánchez-López M. Pozuelo-Carrascosa D. P. Martínez-Vizcaíno V. (2020). Aerobic fitness and academic achievement: a systematic review and meta-analysis. J. Sports Sci. 38, 582–589. doi: 10.1080/02640414.2020.1720496, PMID: 32005082

[ref6] Anderson T. Berry N. T. Wideman L. (2019). Exercise and the hypothalamic–pituitary–adrenal axis: a special focus on acute cortisol and growth hormone responses. Curr. Opin. Endocr. Metab. Res. 9, 74–77. doi: 10.1016/j.coemr.2019.08.002

[ref7] Avilés-Martínez M. A. López-Román F. J. Gómez G. de Cádiz M. J. Arnau-Sánchez J. Martínez-Ros M. T.. (2022). Benefits of a community physical exercise program prescribed from primary care for perimenopausal/menopausal women. Aten. Primaria 54:102119. doi: 10.1016/J.APRIM.2021.102119 34634454 PMC8515407

[ref8] Azer S. A. Guerrero A. P. S. Walsh A. (2013). Enhancing learning approaches: practical tips for students and teachers. Med. Teach. 35, 433–443. doi: 10.3109/0142159X.2013.775413, PMID: 23496121

[ref9] Azevedo C. V. Hashiguchi D. Campos H. C. Figueiredo E. V. Otaviano S. F. S. D. Penitente A. R.. (2023). The effects of resistance exercise on cognitive function, amyloidogenesis, and neuroinflammation in Alzheimer’s disease. Front. Neurosci. 17:1131214. doi: 10.3389/fnins.2023.1131214, PMID: 36937673 PMC10017453

[ref10] Barahona-Fuentes G. Ojeda Á. H. Chirosa-Ríos L. (2021). Effects of training with different modes of strength intervention on psychosocial disorders in adolescents: a systematic review and meta-analysis. Int. J. Environ. Res. Public Health 18:9477. doi: 10.3390/IJERPH18189477, PMID: 34574400 PMC8471285

[ref11] Bartholomew J. B. Mitchell N. G. Bibeau W. S. (2011). Effects of a 12-week resistance exercise program on physical self-perceptions in college students. Res. Q. Exerc. Sport 82, 291–301. doi: 10.1080/02701367.2011.10599757, PMID: 21699109

[ref12] Bartolomei S. Grillone G. Di Michele R. Cortesi M. (2021). A comparison between male and female athletes in relative strength and power performances. J. Funct. Morphol. Kinesiol. 6:17. doi: 10.3390/JFMK6010017, PMID: 33572280 PMC7930971

[ref13] Basso J. C. Suzuki W. A. (2017). The effects of acute exercise on mood, cognition, neurophysiology, and neurochemical pathways: a review. Brain Plast 2, 127–152. doi: 10.3233/BPL-160040, PMID: 29765853 PMC5928534

[ref14] Becker L. Semmlinger L. Rohleder N. (2021). Resistance training as an acute stressor in healthy young men: associations with heart rate variability, alpha-amylase, and cortisol levels. Stress 24, 318–330. doi: 10.1080/10253890.2020.1799193, PMID: 32744460

[ref15] Behm D. G. Faigenbaum A. D. Falk B. Klentrou P. (2008). Canadian Society for Exercise Physiology position paper: resistance training in children and adolescents. Appl. Physiol. Nutr. Metab. 33, 547–561. doi: 10.1139/H08-020, PMID: 18461111

[ref16] Behringer M. Vom Heede A. Yue Z. Mester J. (2010). Effects of resistance training in children and adolescents: a meta-analysis. Pediatrics 126, e1199–e1210. doi: 10.1542/PEDS.2010-0445 20974785

[ref17] Bellido D. Bellido V. (2016). Composición corporal en niños y adolescentes: en búsqueda de la técnica ideal. Nutr. Hosp. 33, 1013–1014. doi: 10.20960/NH.560, PMID: 27759964

[ref18] Bhimani R. Punjani B. Peden-McAlpine C. (2021). Understanding clinical characteristics of muscle weakness. J. Neurosci. Nurs. 53, 69–74. doi: 10.1097/JNN.0000000000000574 33538459

[ref19] Blomstrand P. Tesan D. Nylander E. M. Ramstrand N. (2023). Mind body exercise improves cognitive function more than aerobic- and resistance exercise in healthy adults aged 55 years and older – an umbrella review. Eur. Rev. Aging Phys. Act. 20:15. doi: 10.1186/S11556-023-00325-4, PMID: 37558977 PMC10413530

[ref20] Boytar A. N. Skinner T. L. Wallen R. E. Jenkins D. G. Dekker Nitert M. (2023). The effect of exercise prescription on the human gut microbiota and comparison between clinical and apparently healthy populations: a systematic review. Nutrients 15:1534. doi: 10.3390/nu15061534, PMID: 36986264 PMC10054511

[ref21] Brooks M. A. Edwards N. M. Faigenbaum A. D. (2022). Misconceptions about youth weight lifting. JAMA Pediatr. 176, 1051–1052. doi: 10.1001/JAMAPEDIATRICS.2022.3063, PMID: 36036915

[ref22] Brush C. J. Olson R. L. Ehmann P. J. Osovsky S. Alderman B. L. (2016). Dose-response and time course effects of acute resistance exercise on executive function. J. Sport Exerc. Psychol. 38, 396–408. doi: 10.1123/jsep.2016-0027, PMID: 27385719

[ref23] Burtscher J. Millet G. P. Place N. Kayser B. Zanou N. (2021). The muscle-brain Axis and neurodegenerative diseases: the key role of mitochondria in exercise-induced neuroprotection. Int. J. Mol. Sci. 22:6479. doi: 10.3390/IJMS22126479, PMID: 34204228 PMC8235687

[ref24] Cadenas-Sanchez C. Migueles J. H. Esteban-Cornejo I. Mora-Gonzalez J. Henriksson P. Rodriguez-Ayllon M.. (2020). Fitness, physical activity and academic achievement in overweight/obese children. J. Sports Sci. 38, 731–740. doi: 10.1080/02640414.2020.1729516, PMID: 32091309

[ref25] Calatayud J. Vinstrup J. Jakobsen M. D. Sundstrup E. Brandt M. Jay K.. (2016). Importance of mind-muscle connection during progressive resistance training. Eur. J. Appl. Physiol. 116, 527–533. doi: 10.1007/S00421-015-3305-7, PMID: 26700744

[ref26] Calestine J. BOPP M. BOPP C. M. PAPALIA Z. (2017). College student work habits are related to physical activity and fitness. Int. J. Exerc. Sci. 10:1009–1017. 29170702 10.70252/XLOM8139 PMC5685070

[ref27] Cano Sokoloff N. Misra M. Ackerman K. E. (2016). Exercise, training, and the hypothalamic-pituitary-gonadal Axis in men and women. Front. Horm. Res. 47:27. doi: 10.1159/000445154, PMID: 27348623 PMC7043068

[ref28] Cappelen A. W. Charness G. Ekström M. Gneezy U. Tungodden B. (2017). Exercise improves academic performance. SSRN Electron. J. doi: 10.2139/SSRN.3033774

[ref29] Celis-Morales C. A. Welsh P. Lyall D. M. Steell L. Petermann F. Anderson J.. (2018). Associations of grip strength with cardiovascular, respiratory, and cancer outcomes and all cause mortality: prospective cohort study of half a million UK biobank participants. BMJ 361:1651. doi: 10.1136/BMJ.K1651 PMC5939721 29739772

[ref30] Cheng A. Zhao Z. Liu H. Yang J. Luo J. (2022). The physiological mechanism and effect of resistance exercise on cognitive function in the elderly people. Front. Public Heal. 10:1013734. doi: 10.3389/fpubh.2022.1013734, PMID: 36483263 PMC9723356

[ref31] Collins H. Booth J. N. Duncan A. Fawkner S. Niven A. (2019). The effect of resistance training interventions on ‘the self’ in youth: a systematic review and Meta-analysis. Sport. Med. Open 5:29. doi: 10.1186/S40798-019-0205-0, PMID: 31270635 PMC6609926

[ref32] Dahab K. S. McCambridge T. M. (2009). Strength training in children and adolescents: raising the Bar for Young athletes? Sports Health 1, 223–226. doi: 10.1177/1941738109334215, PMID: 23015875 PMC3445252

[ref33] Damluji A. A. Alfaraidhy M. AlHajri N. Rohant N. N. Kumar M. Al Malouf C.. (2023). Sarcopenia and cardiovascular diseases. Circulation 147, 1534–1553. doi: 10.1161/CIRCULATIONAHA.123.064071, PMID: 37186680 PMC10180053

[ref34] De Prada E. Mareque M. Pino-Juste M. (2022). Teamwork skills in higher education: is university training contributing to their mastery? Psicol Reflexão e Crítica Rev. Semest. do Dep. Psicol. da UFRGS 35:207. doi: 10.1186/S41155-022-00207-1 PMC8828815 35141845

[ref35] Desai N. Vance D. D. Rosenwasser M. P. Ahmad C. S. (2019). Artistic gymnastics injuries; epidemiology, evaluation, and treatment. J. Am. Acad. Orthop. Surg. 27, 459–467. doi: 10.5435/JAAOS-D-18-00147 31232791

[ref36] Devaux M. Vuik S. (2019). The relationship between childhood obesity and educational outcomes | The Heavy Burden of Obesity: The Economics of Prevention | OECD iLibrary. The Heavy Burden of Obesity. Available at: https://www.oecd-ilibrary.org/sites/641a2e79-en/index.html?itemId=/content/component/641a2e79-en (Accessed December 31, 2023)

[ref37] Di Bartolomeo G. Papa S. (2019). The effects of physical activity on social interactions: the case of trust and trustworthiness. J. Sports Econom. 20, 50–71. doi: 10.1177/1527002517717299

[ref38] Du Toit D. Pienaar A. E. Truter L. (2011). Relationship between physical fitness and academic performance in south African children. South African J. Res. Sport. Phys. Educ. Recreat. 33, 23–35.

[ref39] Esteban-Cornejo I. Martinez-Gomez D. Gómez-Martínez S. del Campo-Vecino J. Fernández-Santos J. Castro-Piñero J.. (2016). Inflammatory biomarkers and academic performance in youth. The UP & DOWN study. Brain Behav. Immun. 54, 122–127. doi: 10.1016/J.BBI.2016.01.010, PMID: 26778777

[ref40] Faigenbaum A. D. Kraemer W. J. Blimkie C. J. R. Jeffreys I. Micheli L. J. Nitka M.. (2009). Youth resistance training: updated position statement paper from the national strength and conditioning association. J. Strength Cond. Res. 23, S60–S79. doi: 10.1519/JSC.0B013E31819DF407, PMID: 19620931

[ref41] Faigenbaum A. D. Myer G. D. (2010). Pediatric resistance training: benefits, concerns, and program design considerations. Curr. Sports Med. Rep. 9, 161–168. doi: 10.1249/JSR.0B013E3181DE1214 20463500

[ref42] Ferland P. M. Comtois A. S. (2019). Classic powerlifting performance: a systematic review. J. Strength Cond. Res. 33, S194–S201. doi: 10.1519/JSC.0000000000003099, PMID: 30844981

[ref43] Fisberg M. Maximino P. Kain J. Kovalskys I. (2016). Obesogenic environment – intervention opportunities. J. Pediatr. 92, S30–S39. doi: 10.1016/J.JPED.2016.02.007 27005593

[ref44] Fisher J. (2013). A critical commentary on the practical application of resistance training studies. J. Trainol. 2, 10–12. doi: 10.17338/TRAINOLOGY.2.2_10

[ref45] Fortunato A. K. Pontes W. M. De Souza D. M. S. Prazeres J. S. F. Marcucci-Barbosa L. S. Santos J. M. M.. (2018). Strength training session induces important changes on physiological, immunological, and inflammatory biomarkers. J Immunol Res 2018, 1–12. doi: 10.1155/2018/9675216, PMID: 30046617 PMC6038656

[ref46] Fraile-Martinez O. Alvarez-Mon M. A. Garcia-Montero C. Pekarek L. Guijarro L. G. Lahera G.. (2022). Understanding the basis of major depressive disorder in oncological patients: biological links, clinical management, challenges, and lifestyle medicine. Front. Oncol. 12:956923. doi: 10.3389/FONC.2022.956923, PMID: 36185233 PMC9524231

[ref47] García-Baños C. Rubio-Arias J. Á. Martínez-Aranda L. M. Ramos-Campo D. J. (2020, 2020). Secondary-school-based interventions to improve muscular strength in adolescents: a systematic review. Sustain. For. 12:6814. doi: 10.3390/SU12176814

[ref48] Goldfield G. S. Kenny G. P. Alberga A. S. Prud'homme D. Hadjiyannakis S. Gougeon R.. (2015). Effects of aerobic training, resistance training, or both on psychological health in adolescents with obesity: the HEARTY randomized controlled trial. J. Consult. Clin. Psychol. 83, 1123–1135. doi: 10.1037/CCP0000038 26322787

[ref49] Gordon B. R. McDowell C. P. Hallgren M. Meyer J. D. Lyons M. Herring M. P. (2018). Association of Efficacy of resistance exercise training with depressive symptoms: Meta-analysis and Meta-regression analysis of randomized clinical trials. JAMA Psychiatry 75, 566–576. doi: 10.1001/JAMAPSYCHIATRY.2018.0572, PMID: 29800984 PMC6137526

[ref50] Gouveia É. R. Gouveia B. R. Marques A. Lopes H. Rodrigues A. Peralta M.. (2020). Physical fitness predicts subsequent improvement in academic achievement: differential patterns depending on pupils’ age. Sustain. For. 12:8874. doi: 10.3390/SU12218874

[ref51] Haapala E. (2022). Physical activity, academic performance and cognition in children and adolescents. A systematic review. Balt. J. Heal. Phys. Act 4:7. doi: 10.2478/v10131-012-0007-y

[ref52] Hall F. C. Noonan R. J. (2023). A qualitative study of how and why gym-based resistance training may benefit women’s mental health and wellbeing. Perform. Enhanc. Heal. 11:100254. doi: 10.1016/J.PEH.2023.100254

[ref53] Hart E. Meehan W. P. Bae D. S. D’Hemecourt P. Stracciolini A. (2018). The Young injured gymnast: a literature review and discussion. Curr. Sports Med. Rep. 17, 366–375. doi: 10.1249/JSR.0000000000000536, PMID: 30407945

[ref54] Hinds J. A. Sanchez E. R. (2022). The role of the hypothalamus–pituitary–adrenal (HPA) Axis in test-induced anxiety: assessments, physiological responses, and molecular details. Stress 2, 146–155. doi: 10.3390/STRESSES2010011

[ref55] Hollingsworth J. C. Young K. C. Abdullah S. F. Wadsworth D. D. Abukhader A. Elfenbein B.. (2020). Protocol for minute calisthenics: a randomized controlled study of a daily, habit-based, bodyweight resistance training program. BMC Public Health 20:1242. doi: 10.1186/S12889-020-09355-4, PMID: 32799849 PMC7429724

[ref56] Holmes M. E. Kvasnicka M. A. Brocato D. K. Webb H. E. (2018). Metabolic health and academic achievement in youth at risk for high school dropout in rural Mississippi: the role of classroom management. Prev. Med. Reports 11, 115–119. doi: 10.1016/J.PMEDR.2018.06.003, PMID: 29992081 PMC6038109

[ref57] Hötting K. Schickert N. Kaiser J. Röder B. Schmidt-Kassow M. (2016). The effects of acute physical exercise on memory, peripheral BDNF, and cortisol in Young adults. Neural Plast. 2016, 6860573–6860512. doi: 10.1155/2016/6860573, PMID: 27437149 PMC4942640

[ref58] Hunter S. K. (2014). Sex differences in human fatigability: mechanisms and insight to physiological responses. Acta Physiol (Oxf.) 210, 768–789. doi: 10.1111/APHA.12234, PMID: 24433272 PMC4111134

[ref59] Hunter S. K. (2016). The relevance of sex differences in performance fatigability. Med. Sci. Sports Exerc. 48, 2247–2256. doi: 10.1249/MSS.0000000000000928, PMID: 27015385 PMC5349856

[ref60] Hurley K. S. FLIPPIN K. J. BLOM L. C. BOLIN J. E. HOOVER D. L. JUDGE L. W. (2018). Practices, perceived benefits, and barriers to resistance training among women enrolled in college. Int. J. Exerc. Sci. 11:226. 29795737 10.70252/ZRMT3507 PMC5955292

[ref61] Innocent S. Opiyo Andala D. H. (2021). Relationship between students’ discipline and academic performance in secondary schools in Rwanda. J. Educ. 4, 20–37. doi: 10.53819/810181025021

[ref62] Ishihara T. Morita N. Nakajima T. Yamatsu K. Okita K. Sagawa M.. (2021). Differential effects of changes in cardiorespiratory fitness on worst- and best- school subjects. npj Sci. Learn 6, 1–6. doi: 10.1038/s41539-021-00086-8, PMID: 33795680 PMC8016962

[ref63] Jebeile H. Kelly A. S. O’Malley G. Baur L. A. (2022). Obesity in children and adolescents: epidemiology, causes, assessment, and management. Lancet Diabetes Endocrinol. 10, 351–365. doi: 10.1016/S2213-8587(22)00047-X, PMID: 35248172 PMC9831747

[ref64] Jeffries V. Salzer M. S. (2022). Mental health symptoms and academic achievement factors. J. Am. Coll. Heal. 70, 2262–2265. doi: 10.1080/07448481.2020.1865377 33513071

[ref65] Judge L. W. Burke J. R. (2010). The effect of recovery time on strength performance following a high-intensity bench press workout in males and females. Int. J. Sports Physiol. Perform. 5, 184–196. doi: 10.1123/IJSPP.5.2.184, PMID: 20625191

[ref66] Kamp C. F. Sperlich B. Holmberg H. C. (2014). Exercise reduces the symptoms of attention-deficit/hyperactivity disorder and improves social behaviour, motor skills, strength and neuropsychological parameters. Acta Paediatr. 103, 709–714. doi: 10.1111/APA.12628, PMID: 24612421

[ref67] Kazeminasab F. Sharafifard F. Miraghajani M. Behzadnejad N. Rosenkranz S. K. (2023). The effects of exercise training on insulin resistance in children and adolescents with overweight or obesity: a systematic review and meta-analysis. Front. Endocrinol. 14:1178376. doi: 10.3389/FENDO.2023.1178376/BIBTEX PMC10450243 37635963

[ref68] Keating X. D. Castelli D. Ayers S. F. (2013). Association of weekly strength exercise frequency and academic performance among students at a large university in the United States. J. Strength Cond. Res. 27, 1988–1993. doi: 10.1519/JSC.0B013E318276BB4C, PMID: 23096065

[ref69] Kelso A. Linder S. Reimers A. K. Klug S. J. Alesi M. Scifo L.. (2020). Effects of school-based interventions on motivation towards physical activity in children and adolescents: a systematic review and meta-analysis. Psychol. Sport Exerc. 51:101770. doi: 10.1016/J.PSYCHSPORT.2020.101770

[ref70] Kohl H. W. III Cook H. D. (2013). Physical Activity, Fitness, and Physical Education: Effects on Academic Performance. Available at: https://www.ncbi.nlm.nih.gov/books/NBK201501/ (Accessed December 31, 2023).

[ref71] Kristjánsson Á. L. Sigfúsdóttir I. D. Allegrante J. P. (2010). Health behavior and academic achievement among adolescents: the relative contribution of dietary habits, physical activity, body mass index, and self-esteem. Health Educ. Behav. 37, 51–64. doi: 10.1177/1090198107313481, PMID: 18541647

[ref72] Kuo-Ming W. Peng-sheng W. Yi-ching H. (2012). Physical fitness and academic achievement of elementary school students: a cross-sectional survey in southern Taiwan. J. Phys. Educ. Sport 12, 302–309. doi: 10.7752/jpes.2012.03045

[ref73] Landry B. W. Driscoll S. W. (2012). Physical activity in children and adolescents. PM R 4, 826–832. doi: 10.1016/J.PMRJ.2012.09.585 23174545

[ref74] Lapidot Y. Maya M. Reshef L. Cohen D. Ornoy A. Gophna U.. (2023). Relationships of the gut microbiome with cognitive development among healthy school-age children. Front. Pediatr. 11:1198792. doi: 10.3389/fped.2023.1198792, PMID: 37274812 PMC10235814

[ref75] Liu S. Yu Q. Li Z. Cunha P. M. Zhang Y. Kong Z.. (2020). Effects of acute and chronic exercises on executive function in children and adolescents: a systemic review and meta-analysis. Front. Psychol. 11:554915. doi: 10.3389/fpsyg.2020.554915, PMID: 33391074 PMC7773601

[ref76] Lloyd R. S. Faigenbaum A. D. Stone M. H. Oliver J. L. Jeffreys I. Moody J. A.. (2014). Position statement on youth resistance training: the 2014 international consensus. Br. J. Sports Med. 48, 498–505. doi: 10.1136/BJSPORTS-2013-092952, PMID: 24055781

[ref77] Lőrincz H. Somodi S. Ratku B. Harangi M. Paragh G. (2023). Crucial regulatory role of Organokines in relation to metabolic changes in non-diabetic obesity. Meta 13:270. doi: 10.3390/METABO13020270, PMID: 36837889 PMC9967669

[ref78] Maestroni L. Read P. Bishop C. Papadopoulos K. Suchomel T. J. Comfort P.. (2020). The benefits of strength training on musculoskeletal system health: practical applications for interdisciplinary care. Sport. Med. 50, 1431–1450. doi: 10.1007/s40279-020-01309-5, PMID: 32564299

[ref79] Martin A. J. Steinbeck K. (2017). The role of puberty in students’ academic motivation and achievement. Learn. Individ. Differ. 53, 37–46. doi: 10.1016/J.LINDIF.2016.11.003

[ref80] Mathew J. S. (2017). Self-perception and academic achievement. Indian J. Sci. Technol. 10, 1–6. doi: 10.17485/ijst/2017/v10i14/107586

[ref81] Mattson M. P. (2008). Hormesis defined. Ageing Res. Rev. 7, 1–7. doi: 10.1016/J.ARR.2007.08.007, PMID: 18162444 PMC2248601

[ref82] Mcewan D. Ruissen G. R. Eys M. A. Zumbo B. D. Beauchamp M. R. (2017). The effectiveness of teamwork training on teamwork behaviors and team performance: a systematic review and Meta-analysis of controlled interventions. PLoS One 12:e0169604. doi: 10.1371/JOURNAL.PONE.0169604, PMID: 28085922 PMC5234826

[ref83] Mear E. Gladwell V. F. Pethick J. (2022). The effect of breaking up sedentary time with calisthenics on neuromuscular function: a preliminary study. Int. J. Environ. Res. Public Health 19:14597. doi: 10.3390/IJERPH192114597, PMID: 36361476 PMC9653850

[ref84] Miller M. G. Cheatham C. C. Patel N. D. (2010). Resistance training for adolescents. Pediatr. Clin. N. Am. 57, 671–682. doi: 10.1016/J.PCL.2010.02.009 20538150

[ref85] Montoro M. V. P. Montilla J. A. P. Aguilera E. L. Checa M. A. (2015). Sarcopenia intervention with progressive resistance training and protein nutritional supplements. Nutr. Hosp. 31, 1481–1490. doi: 10.3305/NH.2015.31.4.8489 25795931

[ref86] Myers A. M. Beam N. W. Fakhoury J. D. (2017). Resistance training for children and adolescents. Transl. Pediatr. 6, 137–143. doi: 10.21037/TP.2017.04.01, PMID: 28795003 PMC5532191

[ref87] Nava-Bringas T. I. López-Domínguez L. Macías-Hernández S. I. Espinosa-Morales R. Chávez-Arias D. D. Coronado-Zarco R. (2018). Asociación de la composición corporal total con la fuerza del tronco, el dolor y la discapacidad en pacientes con espondiloartrosis lumbar. Cir. Cir. 86, 388–391. doi: 10.24875/CIRU.18000006, PMID: 30226492

[ref88] Nuzzo J. L. (2020). Sex difference in participation in muscle-strengthening activities. J. Lifestyle Med. 10, 110–115. doi: 10.15280/JLM.2020.10.2.110, PMID: 32995338 PMC7502892

[ref89] Ortega M. A. Fraile-Martínez O. García-Montero C. Pekarek L. Guijarro L. G. Castellanos A. J.. (2021). Physical activity as an imperative support in breast cancer management. Cancers (Basel) 13, 1–30. doi: 10.3390/cancers13010055, PMID: 33379177 PMC7796347

[ref90] Paredes L. Obando I. Leal M. Alvarez C. (2021). Adiposity and muscle strength level in pre-scholars according to the educational level and socio-demographic characteristics of their parents. Andes Pediatr. Rev. Chil. Pediatr. 92, 193–201. doi: 10.32641/ANDESPEDIATR.V92I2.1498, PMID: 34106157

[ref91] Peake J. M. Markworth J. F. Nosaka K. Raastad T. Wadley G. D. Coffey V. G. (2015). Modulating exercise-induced hormesis: Does less equal more? J Appl Physiol (1985) 119, 172–189. doi: 10.1152/JAPPLPHYSIOL.01055.2014, PMID: 25977451

[ref92] Peña J. Yanez C. Gomez C. Martin W. Castillo C. Granados J.. (2019). The relationship between strength and academic performance: a new reason to promote physical activity. Eur. J. Pub. Health 29:429. doi: 10.1093/EURPUB/CKZ186.429

[ref93] Phillips S. M. Winett R. A. (2010). Uncomplicated resistance training and health-related outcomes: evidence for a public health mandate. Curr. Sports Med. Rep. 9, 208–213. doi: 10.1249/JSR.0B013E3181E7DA73, PMID: 20622538 PMC4086449

[ref94] Pochetti J. Ponczosznik D. Filártiga P. R. Testa N. (2018). Strength training in children and adolescents: benefits, risks and recommendations. Arch. Argent. Pediatr. 116, S82–S91. doi: 10.5546/AAP.2018.S82, PMID: 30525318

[ref95] Polevoy G. (2022). The influence of speed and strength training at school on the indicators of attention switching in children aged 13-14 years with different typologies. J. Educ. Health Promot. 11:23. doi: 10.4103/JEHP.JEHP_413_21, PMID: 35281404 PMC8893077

[ref96] Rai M. Demontis F. (2022). Muscle-to-brain signaling via Myokines and Myometabolites. Brain Plast. 8, 43–63. doi: 10.3233/BPL-210133, PMID: 36448045 PMC9661353

[ref97] Ramírez-Vélez R. Garcia-Hermoso A. Prieto-Benavides D. H. Correa-Bautista J. E. Quino-Ávila A. C. Rubio-Barreto C. M.. (2019). Muscle mass to visceral fat ratio is an important predictor of the metabolic syndrome in college students. Br. J. Nutr. 121, 330–339. doi: 10.1017/S0007114518003392, PMID: 30556511

[ref98] Reuter P. R. Forster B. L. (2021). Student health behavior and academic performance. PeerJ 9:e11107. doi: 10.7717/peerj.11107, PMID: 33959411 PMC8054760

[ref99] Roberts B. M. Nuckols G. Krieger J. W. (2020). Sex differences in resistance training: a systematic review and Meta-analysis. J. Strength Cond. Res. 34, 1448–1460. doi: 10.1519/JSC.0000000000003521 32218059

[ref100] Robinson K. Riley N. Owen K. Drew R. Mavilidi M. F. Hillman C. H.. (2023). Effects of resistance training on academic outcomes in school-aged youth: a systematic review and meta-analysis. Sports Med. 53, 2095–2109. doi: 10.1007/S40279-023-01881-6, PMID: 37466900 PMC10587249

[ref101] Rosenthal M. McPherson A. M. Docherty C. L. Klossner J. (2021). Perceptions and utilization of strength training and conditioning in collegiate contemporary and ballet dancers: a qualitative approach. Med. Probl. Perform. Art. 36, 78–87. doi: 10.21091/MPPA.2021.2012, PMID: 34079981

[ref102] Salas E. DiazGranados D. Klein C. Burke C. S. Stagl K. C. Goodwin G. F.. (2008). Does team training improve team performance? A meta-analysis. Hum. Factors 50, 903–933. doi: 10.1518/001872008X375009 19292013

[ref103] Salimans L. Liberman K. Njemini R. Kortekaas Krohn I. Gutermuth J. Bautmans I. (2022). The effect of resistance exercise on the immune cell function in humans: a systematic review. Exp. Gerontol. 164:111822. doi: 10.1016/J.EXGER.2022.111822, PMID: 35490790

[ref104] Sánchez-Hernando B. Antón-Solanas I. Juárez-Vela R. Gea-Caballero V. Carboneres-Tafaner M. I. Ferrer-Gracia E.. (2021). Healthy lifestyle and academic performance in middle school students from the region of Aragón (Spain). Int. J. Environ. Res. Public Health 18:8624. doi: 10.3390/IJERPH18168624, PMID: 34444372 PMC8393534

[ref105] Sardinha L. B. Marques A. Minderico C. Palmeira A. Martins S. Santos D. A.. (2016). Longitudinal relationship between cardiorespiratory fitness and academic achievement. Med. Sci. Sports Exerc. 48, 839–844. doi: 10.1249/MSS.0000000000000830, PMID: 26606272 PMC6258904

[ref106] Shi P. Tang Y. Zhang Z. Feng X. Li C. (2022). Effect of physical exercise in real-world settings on executive function of typical children and adolescents: a systematic review. Brain Sci. 12:1734. doi: 10.3390/brainsci12121734, PMID: 36552193 PMC9775424

[ref107] Silverman M. N. Deuster P. A. (2014). Biological mechanisms underlying the role of physical fitness in health and resilience. Interface Focus 4:20140040. doi: 10.1098/RSFS.2014.0040, PMID: 25285199 PMC4142018

[ref108] Simba N. O. Agak J. O. Kabuka E. K. (2016). Impact of discipline on academic performance of pupils in public primary schools in Muhoroni Sub-County, Kenya. J. Educ. Pract. 7, 164–173.

[ref109] Strickland J. C. Smith M. A. (2014). The anxiolytic effects of resistance exercise. Front. Psychol. 5:753. doi: 10.3389/FPSYG.2014.00753, PMID: 25071694 PMC4090891

[ref110] Suchomel T. J. Nimphius S. Bellon C. R. Stone M. H. (2018). The importance of muscular strength: training considerations. Sport. Med 484, 765–785. doi: 10.1007/S40279-018-0862-Z 29372481

[ref9001] Smail K. Horvat M. (2006). Relationship of muscular strength on work performance in high school students with mental retardation. Educ. Train. Dev. Disabil, 41, 410–419.

[ref111] Trudeau F. Shephard R. J. (2009). Relationships of physical activity to brain health and the academic performance of schoolchildren. Am. J. Lifestyle Med. 4, 138–150. doi: 10.1177/1559827609351133

[ref112] Van Dusen D. P. Kelder S. H. Kohl H. W. Ranjit N. Perry C. L. (2011). Associations of physical fitness and academic performance among schoolchildren. J. Sch. Health 81, 733–740. doi: 10.1111/J.1746-1561.2011.00652.X 22070504

[ref113] Villarrasa-Sapiña I. Álvarez-Pitti J. Cabeza-Ruiz R. Redón P. Lurbe E. García-Massó X. (2018). Relationship between body composition and postural control in prepubertal overweight/obese children: a cross-sectional study. Clin. Biomech 52, 1–6. doi: 10.1016/J.CLINBIOMECH.2017.12.010, PMID: 29291461

[ref114] Wick K. Kriemler S. Granacher U. (2021). Effects of a strength-dominated exercise program on physical fitness and cognitive performance in preschool children. J. Strength Cond. Res. 35, 983–990. doi: 10.1519/JSC.0000000000003942, PMID: 33752222

[ref115] Winwood P. W. Cronin J. B. Posthumus L. R. Finlayson S. J. Gill N. D. Keogh J. W. L. (2015). Strongman vs. traditional resistance training effects on muscular function and performance. J. Strength Cond. Res. 29, 429–439. doi: 10.1519/JSC.0000000000000629, PMID: 25627449

